# Nurse-Like Cells and Chronic Lymphocytic Leukemia B Cells: A Mutualistic Crosstalk inside Tissue Microenvironments

**DOI:** 10.3390/cells10020217

**Published:** 2021-01-22

**Authors:** Stefania Fiorcari, Rossana Maffei, Claudio Giacinto Atene, Leonardo Potenza, Mario Luppi, Roberto Marasca

**Affiliations:** 1 Department of Medical and Surgical Sciences, Section of Hematology, University of Modena and Reggio Emilia, 41124 Modena, Italy; rossana.maffei@unimore.it (R.M.); claatene@unimore.it (C.G.A.); leonardo.potenza@unimore.it (L.P.); mario.luppi@unimore.it (M.L.); 2 Hematology Unit, Department of Oncology and Hematology, A.O.U of Modena, Policlinico, 41124 Modena, Italy

**Keywords:** CLL, microenvironment, nurse-like cells

## Abstract

Chronic lymphocytic leukemia (CLL) is the most common adult leukemia in Western countries and is an example of hematological disease where cooperation between genetic defects and tumor microenvironmental interaction is involved in pathogenesis. CLL is a disease that is considered as “addicted to the host”; indeed, the crosstalk between leukemic cells and the tumor microenvironment is essential for leukemic clone maintenance supporting CLL cells’ survival, proliferation, and protection from drug-induced apoptosis. CLL cells are not innocent bystanders but actively model and manipulate the surrounding microenvironment to their own advantage. Besides the different players involved in this crosstalk, nurse-like cells (NLC) resemble features related to leukemia-associated macrophages with an important function in preserving CLL cell survival and supporting an immunosuppressive microenvironment. This review provides a comprehensive overview of the role played by NLC in creating a nurturing and permissive milieu for CLL cells, illustrating the therapeutic possibilities in order to specifically target and re-educate them.

## 1. Introduction

Chronic lymphocytic leukemia (CLL) is the most common leukemia in adults in Western countries and predominantly affects the elderly, with a median age at diagnosis of 72 years. CLL is characterized by the accumulation of clonal mature CD5^+^ B cells in peripheral blood, bone marrow, and secondary lymphoid organs. The diagnosis of CLL is established by testing the presence of ≥5 × 10^9^/L monoclonal B lymphocytes in the peripheral blood. In the absence of lymphadenopathy, organomegaly, cytopenia, and clinical symptoms, the presence of <5 × 10^9^/L monoclonal B lymphocytes defines “monoclonal B lymphocytosis” (MBL), which progresses to CLL in at least 1–2% of MBL cases per year [1].

From a biological and clinical perspective, CLL displays a wide degree of heterogeneity, ranging from patients characterized by a stable disease with a nearly normal life expectancy to patients with an aggressive disease with frequent relapses or transformation into an aggressive lymphoma, such as diffuse large B-cell lymphoma (DLBCL) (Richter transformation). Patients affected by CLL have rising lymphocytosis, adenopathy, hepatosplenomegaly, and bone marrow infiltration, resulting in bone marrow failure with anemia and thrombocytopenia [2]. The reason for such clinical heterogeneity has not been completely elucidated; it is mainly determined by genetic factors and the complex relationship that leukemic cells have with the surrounding microenvironment. CLL is characterized by a distinct immunophenotype with the co-expression of CD19, CD5, and CD23 and low levels of surface immunoglobulins. Studies conducted on the B-cell receptor (BCR) have led to identifying two molecular subgroups: those harboring unmutated immunoglobulin heavy-chain variable region (IGHV) genes (U-CLL, ≥98% identity with the germline) and those with mutated IGHV genes (M-CLL). U-CLL originates from B cells that have not experienced the germinal center reactions, whereas M-CLL originates from post-germinal center B cells [3]. Unmutated IGHV genes predict worse prognosis, and this is possibly due to the enrichment of some genetic lesions that confer high aggressiveness (such as mutations of NOTCH1) and also the predisposition of U-CLL to undergo clonal evolution [2]. In addition, U-CLL cells show a higher rate of in vitro spontaneous apoptosis and are more dependent on the surrounding environment as compared to CLL cells isolated from M-CLL patients [4]. Noteworthy, the BCR of almost 30% of CLL patients displays nearly identical or highly homologous complementarity determining region 3 (CDR3), also known as “stereotyped” BCR, suggesting an antigen-driven path to CLL development. Of note, this feature of stereotypy implies shared somatic mutations, a similar genetic profile of the leukemic clone, and also similar antigen-binding properties and functional responses through the BCR and similar clinical outcomes [5].

Historically, CLL has been considered as an accumulative disease of B lymphocytes with defects in apoptosis that morphologically resemble small resting B cells in the blood with limited ability to proliferate [3,6]. Nowadays, the dynamic nature of CLL clones has been defined, showing the cellular proliferation of leukemic clones inside structures called proliferation centers or pseudofollicles frequently found in the lymph nodes and bone marrow of CLL patients [7,8]. In pseudofollicles, specific cellular populations, including T cells, stromal cells, and macrophages, in addition to the endothelial cells and B prolymphocytes/paraimmunoblasts, are present. Inside these structures, CLL cells’ survival and proliferation is preserved through the activation of BCR signaling [9].

### 1.1. Role of Microenvironment in CLL

In 1889, Stephen Paget stated that “when a plant goes to seed, its seeds are carried in all directions; but they can only live and grow if they fall on congenial soil”, proposing that the microenvironment provides a fertile “soil” for tumor cells (“seeds”), leading to their growth [10].

Evidence of microenvironment dependency is related to the knowledge that despite an apparent long life in vivo, CLL cells derived from peripheral blood die spontaneously and rapidly when cultured in in vitro conditions with media supplemented with either autologous or fetal bovine serum [11]. This suggests that extrinsic signals from microenvironmental elements surrounding leukemic cells in vivo are essential to support prolonged CLL cell survival. In bone marrow and secondary lymphatic tissues, CLL cells entertain complex cellular and molecular interactions with the surrounding accessory cells, collectively referred to as the “microenvironment” [12]. Comparison between different anatomic compartments of the disease, such as peripheral blood, bone marrow, and lymph nodes, has underlined the importance of the crosstalk between leukemic cells and bystander non-malignant cells in lymphoid organs to support CLL maintenance [13]. Noteworthy, lymphocytes continually recirculate from blood to tissues and back to the bloodstream again. Trafficking is mediated by transient interactions with the endothelium through adhesion molecules and chemokines that trigger integrin activation, thus inducing firm adhesion and transendothelial migration into tissues where stromal cells guide lymphocyte homing and retention [14]. In CLL, trafficking and homing into the lymph nodes and bone marrow are an essential part of the disease pathogenesis and progression. Inside tissue niches, CLL cells establish a tight and intimate interaction with many cell types such as extracellular matrix, fibroblasts, cells of the innate and adaptive immune response, and vascular endothelial cells (ECs). This bidirectional network is guaranteed by cell-to-cell contact, adhesion molecules, cell surface ligands, chemokines, cytokines, and their corresponding receptors [15]. CLL cells are not innocent bystander but actively model and manipulate the surrounding microenvironment to their own advantage. For this reason, it is thought that the CLL microenvironment is shaped and maintained through a dynamic, interactive coevolution between leukemic and normal accessory cells [12]. The main players inside tissue microenvironments are mesenchymal stromal cells, leukemia-related macrophages, T cells, NK cells, and endothelial cells. This complex crosstalk is crucial for CLL cells’ survival and proliferation signals and makes a critical contribution to disease progression and drug resistance or disease relapse [16,17,18,19,20,21].

Different studies have focused the attention on the influence of bone marrow stromal cells in preventing the apoptosis of CLL cells. Adhesive interaction between them is able to deliver signals which regulate the survival pathways in CLL cells, inducing protection from spontaneous apoptosis and reducing susceptibility to therapeutic agents [22,23]. Among the plethora of factors involved in this crosstalk, VLA-4 integrin (CD49d), on CLL cells plays a critical role through the binding to VCAM-1 (CD106) on the stromal cell surface. VLA-4 is variable expressed by CLL patients and predicts disease progression; indeed, high levels of VLA-4 are associated by a more disease progression [24,25]. Binding of VCAM-1 by VLA-4 is able to confer protection to CLL cells from apoptosis and drug resistance [26]. Again, CD40 is expressed on CLL cells and is able to bind its ligand, CD40L, that is found on activated T lymphocytes. In CLL, the triggering of CD40 induces the survival and proliferation of leukemic cells, counteracting the therapeutic effects of apoptogenic drugs [27,28,29].

Leukemia-cell derived extracellular vesicles represent another possibility of communication with the surrounding microenvironment. Exosomes contain functionally active biological molecules, such as proteins, lipids, mRNA, and microRNA. These molecular structures deliver their content along the bloodstream and lymphatic vessels and release it into the target cells influencing their cellular functions. In CLL, it has been demonstrated that exosomes are able to rescue CLL cells from spontaneous and drug-induced apoptosis, enhance their migration ability, and support the proliferation of leukemic cells [30,31].

BCR activation is critical for CLL maintenance, as its signal transduction pathway is essential for CLL cells’ survival, proliferation, and trafficking. Antigen binding to the BCR activates proximal kinases such as SYK and LYN along with phosphorylation of immunoreceptor tyrosine-based motifs receptor of Igα/Igβ. This activates SYK, BTK, PI3Ks, calcium mobilization, MAP/kinases, phospholipase-C-γ, and NF-κB [32,33]. In particular, BTK is a member of tyrosine protein kinase (Tec) with a critical role in the amplification of the BCR signal. The activation of BTK is involved in the PI3K/Akt pathway and also in the activation of Iκb kinase, which phosphorylates the NF-κB inhibitor I-κBα kinase, allowing NF-κB to translocate to the nucleus [34,35]. The interaction of CLL cells with the tumor microenvironment is controlled by BCR signaling and is involved in the survival and proliferation of leukemic cells [36].

As mentioned, CLL cells are not passive seeds but are able to create supportive conditions aberrantly orchestrating the function of immune effector cells and escaping immunosurveillance. Indeed, CLL cells directly participate to immunomodulation supporting an immunosuppressive environment. The formation of a CLL-induced immunosuppressive milieu includes molecular mechanisms, environmental influence and immune evasion [37]. This is in line with the observation that CLL is characterized by several clinical complications related to alterations in the immune system, including hypogammaglobulinemia, predisposition to infection, and the increased incidence of autoimmune disorders and secondary primary malignancies [38].

### 1.2. Nurse Like Cells

**Figure 1:**
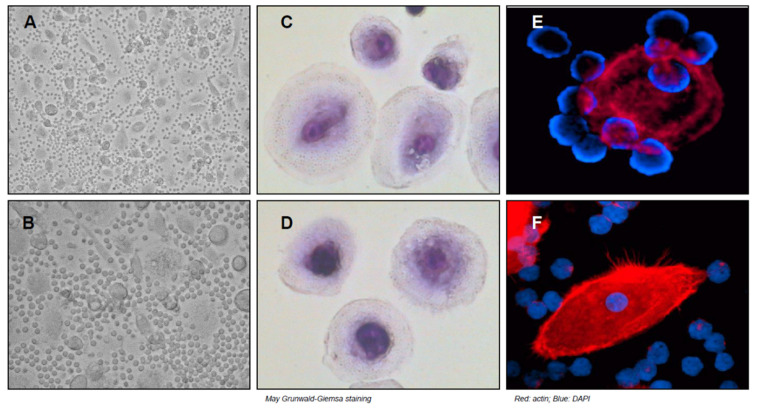
NLC formation from the peripheral blood of patients affected by CLL. PBMC were isolated from CLL patients and cultured in complete medium for 14 days then CLL cells were carefully removed. (**A**–**F**) Figures with phase-contrast microphotographs, May–Grunwald–Giemsa, and immunofluorescence staining show the formation of large and adherent cells known as NLC. As shown, CLL cells are closely attached to NLC.

Inside tissue niches, monocyte/macrophage population assumes a critical role in the maintenance and progression of CLL cells. CLL patients have a high number of circulating monocytes with significant shift toward non-classical population with low level of CD14 together with high CD16 [39]. Non-classical monocytes exhibit patrolling behavior in vivo, are weak phagocytes, and do not produce cytokines in response to cell-surface toll-like receptors [40]. The altered composition and function of blood monocytes in CLL patients could derive from a specific CLL-mediated education of immune cells including an establishment of a skewed phenotype in the monocyte/macrophage population. In tissues infiltrated by CLL cells, it has been identified a specific population of leukemia-associated macrophages called nurse-like cells (NLC). NLC own their name, given important similarities with thymic nurse cells (TNC) that reside in thymic cortex. TNC is a large epithelial cell that contains many viable lymphoid cells within its intracellular vesicles and it provides a microenvironment that is necessary for lymphocyte proliferation and differentiation. Inside TNC, lymphocytes are morphologically intact, and many of them undergo mitosis without any sign of phagocytic degradation. Lymphocytes roll under these cells without been internalized according to a process known as emperipolesis [41,42]. The first observation on the formation of NLC in CLL is thanks to work carried out by Burger et al [43]. The isolation and culture of peripheral mononuclear cells (PBMC) in complete medium for at least 14 days lead to formation of peculiar adherent cells, now known as NLC. (Figure 1) Formation of NLC is closely related to PBMC isolated from CLL patients, indeed culture of PBMC from healthy donors does not lead to formation of NLC unless establishment of a co-culture of CD14^+^ cells of healthy donors with CLL B cells. This strongly suggests the influence of leukemic cells in the generation of NLC. NLC form a monolayer of large, round, and sometimes binucleate cells, with CLL cells firmly attached all around them [43]. NLC have an immunophenotype that distinguishes them from blood circulating monocytes, monocyte-derived dendritic cells, or macrophages. They express at different levels CD14, CD45, HLA-DR, CD33, and CD68 and fail to express CD106, which is usually observed on follicular, dendritic cells marrow and stromal cells. Evidence of NLC’ existence in vivo derives from the culture of CLL splenocytes, which allow the growth of cells very similar to NLC, suggesting that cells with distinctive properties of NLC are present in the secondary lymphoid tissues of CLL patients [18]. In the microenvironment, CLL cells may drive the induction of NLC differentiation through several factors. The enzyme nicotinamide phosphoribosyltransferase (NAMPT) is expressed at high levels by CLL cells and this is also confirmed by measuring extracellular NAMPT at elevated amounts in the plasma of CLL patients. Extracellular NAMPT produced by CLL cells has a pivotal role in polarizing circulating monocytes into macrophages, and blocking NAMPT results in compromising NLC differentiation and phenotype [44]. Moreover, high-mobility group protein B1 (HMGB1) is a nuclear protein that is usually released by damaged cells or dead cells or by immune cells and cancer cells [45]. It has been demonstrated that CLL cells are able to release HMGB1, regulating NLC differentiation through TLR-9/RAGE pathway [46]. Colony-stimulating factor-1 is required for normal homeostasis and survival of macrophages. Mice lacking functional CSF-1 or nullizygous for CSF-1 receptor have a decrease in tissue-resident macrophages [47]. NLC express the CSF-1 receptor, which is important for their generation and survival [48].

### 1.3. Nurturing Properties of NLC

The increased number of NLC in the CLL microenvironment has been associated with disease progression and shorter overall survival [46,49]. The complex interplay between CLL cells and NLC inside tissue niches leads to the protection of CLL cells promoting their recruitment, survival, and proliferation. NLC express high levels of stromal-derived factor 1-α (SDF-1α), a chemokine that is a potent chemoattractant for CLL cells involved in leukemic cells’ migration and emperipolesis [50]. SDF-1α secreted by NLC drives CLL cells inside the protective tissue niches through the activation of the corresponding CXCR4 receptor expressed on the surface of CLL cells. Once leukemic cells are attached to NLC, they are protected from apoptosis through a firm cell-to-cell contact [51]. The engagement of CXCR4 in CLL cells by SDF-1α induces the activation of downstream signaling pathways as MAP kinase and AKT, crucial in the maintenance of CLL cells’ survival [43,52]. In addition, NLC express B cell activation factor (BAFF) and a proliferation-inducing ligand (APRIL) that are both TNF family members, with critical roles in peripheral B cell survival, maturation, and differentiation. BAFF binds to three separate receptors expressed by CLL cells, the BAFF receptor (BAFF-R), the transmembrane activation and calcium modulation ligand interaction (TACI), and the B cell maturation antigen (BCMA). APRIL binds only TACI and BCMA [53]. The stimulation of these receptors induces the activation of NF-κB pathway and the expression of Bcl-2, preserving CLL cells’ survival [54]. In the same line, brain-derived neurotrophic factor (BDNF), secreted by NLC, activates the complex NTSR2-TrkB expressed at the CLL cells’ surface, inducing Src pro-survival signal and the expression of Bcl-2 [55]. Again, NLC release galectin 1 (Gal1) into the surrounding environment. Gal1 is found in the inflammation sites and tumor growth, performing pro-survival effects on malignant cells. Gal1 influences CLL behavior through the modulation of BCR signaling, decreasing the threshold of its activation, or through the control of BAFF and/or APRIL secretion determining opportune microenvironmental conditions for leukemic progression [56]. In response to antigen(s) presented by NLC, BCR activation triggers leading to the increased secretion of chemokines CCL3 and CCL4 by CLL [57]. These chemokines mobilize accessory cells, such as T cells and monocytes, to tissue niches, allowing the formation of a nurturing milieu for CLL cells [58]. As confirmation, gene expression profile analyses conducted on peripheral blood, bone marrow, and lymph nodes samples have demonstrated an inducible, probably antigen-drived BCR signaling in CLL cells in the lymph node that has resulted in Syk and NF-κB activation and the induction of a characteristic gene expression signature [13]. In the CLL microenvironment, secreting and stimulating factors are only partial culprits in the maintenance of leukemic cells’ survival. It has to be considered that CLL cells adhere closely to NLC, establishing firm physical contact. The contact of CLL cells with NLC preserves leukemic cells’ viability through the interaction of LFA-3 (lymphocyte function-associated antigen 3), broadly expressed in leukemic cells that bind CD2 on NLC. The LFA-3/CD2 axis exerts a pro-survival effect on CLL clones and of note LFA-3 expression correlates with increased overall survival after frontline rituximab-based immunochemotherapy [59].

### 1.4. Immunosuppressive Properties of NLC

Cells of the monocyte-macrophage lineage are characterized by diversity and plasticity. In response to different signals, macrophages may be skewed toward classical M1 activation or alternative M2 activation. In particular, M1 polarization is characterized by the expression of high levels of pro-inflammatory cytokines, the production of reactive nitrogen and oxygen intermediates, the promotion of Th1 response, and strong microbial activity. M2 polarized-macrophages promote tissue remodeling and tumor progression and have immunoregulatory functions [60]. CLL patients are characterized by high number of circulating Tie-2-expressing monocytes (TEMs). TEMs are highly proangiogenic subset of myeloid cells in tumors and display immune suppressive activity [61]. In addition, Tie2^+^ NLC are detected in CLL-infiltrated lymph nodes, mainly in a peri-vascular distribution, suggesting that leukemic cells secreting Ang2 in infiltrated-tissue recruit them into tissue from the TEM subpopulation [39,62].

NLC have been considered as CLL-specific tumor-associated macrophages, showing the modulation of genes involved in the regulation of both innate and acquired immunity with impairment in the phagocytic capability [63]. Accordingly, CD163, a marker of macrophages with M2 polarization, is widely expressed in NLC. Its function is carried out through the scavenging of the haptoglobin-hemoglobin complex and the production of anti-inflammatory metabolites [64]. It has been established that the expression of CD163 by NLC correlates with CLL proliferation in lymph nodes and the soluble counterpart of CD163 links with worst prognostic factors, such as TP53 mutations, complex karyotype. and unmutated IGHV. In addition, high levels of CD163 are associated with shorter overall survival and treatment-free survival [49]. The gene expression profiling of NLC shows the dysregulation of genes involved in immunocompetence, with a high expression of CD11b, CD68, CD206, IL-10, CCL-18, and IDO. NLC and CLL monocytes inhibit T-cell proliferation through TGFβ, IL-10, and IDO; in addition, soluble factors produced by NLC drive the expansion of T regulatory cells, reinforcing a link between the impairment of immune response and CLL progression [44,65,66].

### 1.5. NLC as a Therapeutic Target

**Figure 2:**
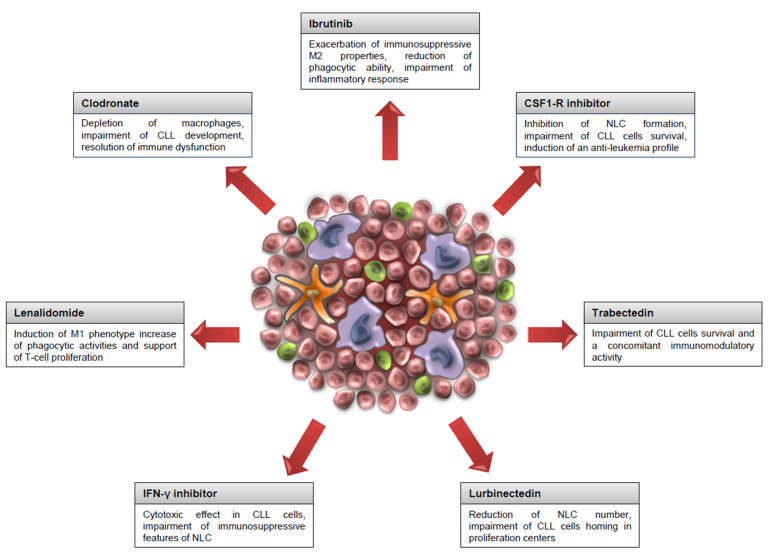
Therapeutic strategies to target NLC. NLC play a critical role in supporting CLL cells’ viability and protecting them from drug-induced apoptosis. In addition, NLC resemble features of M2 macrophages with peculiar immunosuppressive properties. Here, different therapeutic strategies are depicted, showing the possibility to interfere with NLC formation or re-educating them against leukemia.

In the last few years, it has been delineated that NLC do not just nurse but actively participate in the setting of environment-mediated drug resistance and immunosuppression. For this reason, different studies have investigated the rationale of targeting NLC in order to modify their nurturing and protective behavior, looking towards the induction of an immunological re-education (Figure 2). Liposomal clodronate is widely used for the in vivo depletion of macrophages; studies conducted in CLL mice have shown an impairment of CLL development, the resolution of immune dysfunction, and a partial resolution of systemic inflammation [67].

Given the importance of CSF-1 receptors in macrophages’ survival, the inhibition of CSF1-R signaling leads to macrophage depletion in CLL mice, determining their reduction in bone marrow and peripheral blood. Macrophages, targeted via the CSF-1 receptor, shift the CLL microenvironment phenotype toward a more anti-tumor direction, preventing the formation of new macrophages by inducing apoptosis or affecting macrophage differentiation from monocyte precursors and reducing the leukemic cell load [68,69].

Trabectedin is an antitumor agent of marine origin extracted from the tunicate Ecteinascidia turbinata. It acts as a minor groove of the DNA binder that blocks the cell cycle, affects gene transcription, and alters the DNA repair pathway. Trabectedin shows selective cytotoxic effects on monocytes and macrophages, affecting monocyte differentiation to macrophages [70]. In CLL, trabectedin has shown to induce cytotoxic effects in leukemic cells and concomitantly exerts immunomodulatory activity on several cell types of the microenvironment. It depletes myeloid-derived suppressor cells and tumor-associated macrophages and induces T cell response [71].

Lurbinectedin, a trabectedin analogue, is a selective inhibitor of protein-coding genes, leading to arrest the elongation of RNA polymerase II and its degradation by the ubiquitin/proteasome machinery. The recruitment of DNA repair factors determines an accumulation of double-strand breaks, with consequent apoptosis. In CLL, lurbinectedin has a direct effect on leukemic cells and also on the monocyte/macrophage population. This drug indirectly reduces the number of NLC interfering with CLL cells homing in proliferation centers. In addition, lurbinectedin increases the production of IL-1β by monocytes and NLC that is associated with good prognostic markers or increased survival [72]. In order to reprogram NLC, a possibility is the use of interferon γ (IFN-γ). Interferons are considered modulators of macrophage plasticity and activation, in particular, IFN-γ is involved in the promotion of monocyte differentiation. The treatment of NLC with IFN-γ modulates them toward a more effector-like phenotype, concomitantly interfering with CLL cell survival. In the presence of IFN-γ, NLC function as immune effectors with decreased M2 features and increased phagocytic ability [73].

Lenalidomide is an immunomodulatory drug that has been used in CLL. It acts not directly on CLL cells but by modulating the tumor microenvironment. Lenalidomide affects the protection of leukemic cells induced by NLC, inhibiting the migration of CLL cells toward SDF-1α [74]. Furthermore, lenalidomide counteracts the ability of leukemic cells to generate NLC. Instead, it promotes the expansion of a macrophage population with the M1 phenotype characterized by enhanced phagocytic activities and supports T-cell proliferation, with less ability to nurture leukemic cells. Lenalidomide is able to decrease the expression of CD163 and concomitantly induce the activation of CD11b for an effective phagocytosis [75].

Ibrutinib is an irreversible inhibitor of BTK that has demonstrated exceptional efficacy in CLL patients [76,77]. Ibrutinib disrupts the BCR and NF-κB pathways, affecting CLL trafficking, homing, and viability. Despite the impressive improvement in clinical outcomes, some patients show limited benefit from BTK inhibition due to discontinuation due to adverse effects related at least in part to off-target effects related to the tumor microenvironment [78]. Ibrutinib is not able to mobilize NLC from tissues into the bloodstream and supports the protection of NLC to promote CLL survival [79]. Ibrutinib exerts its immunomodulatory effects on NLC through the modulation of BTK expression in this population. BTK in macrophages is involved in the regulation of lineage commitment and the inhibition of BTK in NLC, potentiating the M2-skewed features. Ibrutinib induces the inhibition of phagocytosis, the induction of of M2 markers such as CD163 and CD206, together with negative regulation of M1 polarization; and modulates clusters of genes involved in immune suppression [80].

Recently, invasive fungal infections have been reported among patients receiving treatment with ibrutinib. In this scenario, ibrutinib is able to affect the ability of NLC to counteract *Aspergillus fumigatus* conidia germination due to reduced phagocytosis and the impairment of a productive inflammatory response with a decreased level of IL-1β and TNF-α [81,82].

## 2. Conclusions

CLL is the most common form of adult leukemia in the Western countries. Besides a dynamic landscape of genetic alterations, the disease history is related to the complex intimate crosstalk that leukemic cells entertain with non-malignant accessory cells inside tissue microenvironments. Despite several and important therapeutic advances, nowadays CLL is still an incurable disease and clinical resistance may occur both through the primary biological features of tumor cells or through resistance, which arises through the crosstalk with the surrounding tumor microenvironment. Inside tissue niches, nurse-like cells represent the Achilles’ heel in CLL. In this scenario, NLC are not just caregiver of CLL cells, feeding and protecting them from drugs, but given the complex bidirectional crosstalk, NLC are manipulated and programmed by leukemic cells in order to create an immunosuppressive milieu that allows immune evasion. Therapeutic strategies to directly target NLC, blocking their formation or re-educating these cells against leukemia, need to be envisioned.
